# Accelerating to Zero: Strategies to Eliminate Malaria in the Peruvian Amazon

**DOI:** 10.4269/ajtmh.15-0369

**Published:** 2016-06-01

**Authors:** Antonio M. Quispe, Alejandro Llanos-Cuentas, Hugo Rodriguez, Martin Clendenes, Cesar Cabezas, Luis M. Leon, Raul Chuquiyauri, Marta Moreno, David C. Kaslow, Max Grogl, Sócrates Herrera, Alan J. Magill, Margaret Kosek, Joseph M. Vinetz, Andres G. Lescano, Eduardo Gotuzzo

**Affiliations:** Department of Parasitology, U.S. Naval Medical Research Unit No. 6, Lima, Peru; Department of International Health, Johns Hopkins Bloomberg School of Public Health, Baltimore, Maryland; Instituto de Medicina Tropical Alexander von Humboldt, Universidad Peruana Cayetano Heredia, Lima, Peru; Dirección Regional de Salud Loreto, Ministerio de Salud, Iquitos, Peru; Estrategia Sanitaria Nacional de Enfermedades Metaxénicas, Lima, Peru; Instituto Nacional de Salud, Lima, Peru; Ministerio de Salud, Lima, Peru; Division of Infectious Diseases, Department of Medicine, University of California, San Diego, La Jolla, California; Program for Appropriate Technology in Health, Seattle, Washington; Science Directorship, U.S. Naval Medical Research Unit No. 6, Lima, Peru; Caucaseco Scientific Research Center, Cali, Colombia; Malaria Strategy, Bill & Melinda Gates Foundation, Seattle, Washington; Asociación Benéfica Proyectos de Informática, Salud, Medicina, y Agricultura, Iquitos, Loreto, Peru; School of Public Health and Administration, Universidad Peruana Cayetano Heredia, Lima, Peru

## Abstract

In February 2014, the Malaria Elimination Working Group, in partnership with the Peruvian Ministry of Health (MoH), hosted its first international conference on malaria elimination in Iquitos, Peru. The 2-day meeting gathered 85 malaria experts, including 18 international panelists, 23 stakeholders from different malaria-endemic regions of Peru, and 11 MoH authorities. The main outcome was consensus that implementing a malaria elimination project in the Amazon region is achievable, but would require: 1) a comprehensive strategic plan, 2) the altering of current programmatic guidelines from control toward elimination by including symptomatic as well as asymptomatic individuals for antimalarial therapy and transmission-blocking interventions, and 3) the prioritization of community-based active case detection with proper rapid diagnostic tests to interrupt transmission. Elimination efforts must involve key stakeholders and experts at every level of government and include integrated research activities to evaluate, implement, and tailor sustainable interventions appropriate to the region.

## Introduction

Malaria continues to represent a major public health threat, exerting a significant disease burden worldwide.^1^ Although disease burden is highest in sub-Saharan Africa, malaria also causes a significant burden in other regions such as south and southeast Asia, Oceania, and Central and South America.^2^,^3^ Malaria elimination efforts are primarily focused on African countries and particularly on *Plasmodium falciparum* due to the mortality it causes in the poorest countries in the African region.^4^ This situation has resulted in an increasing gap in the collective knowledge about how to control this disease in regions where the malaria cases are predominantly due to *Plasmodium vivax*,^2^ the most widely distributed *Plasmodium* species that causes malaria.^5^ *Plasmodium vivax* is biologically and epidemiologically different from *P. falciparum* and it is not, therefore, appropriate to assume that interventions developed for falciparum malaria are directly transferable to *P. vivax*.^6^,^7^ With 2.8 billion people at risk of acquiring *P. vivax* malaria,^5^ the question as to how to best interrupt its transmission and achieve elimination in regions where *P. vivax* is highly predominant has received little focused attention. More recent research has demonstrated its potential to be an indirect cause of death,^8^ to adversely impact child growth,^9^ to cause severe and frequent illness,^10^ and to demonstrate a reduced response to chloroquine.^11^,^12^ Given this, malaria researchers have been working to address key knowledge gaps, characterize the pattern of transmission of malaria, and identify high-risk groups that must be targeted to interrupt transmission, as well as to understand the patterns of human migration in vivax-predominant regions.

In the last two decades, research conducted in Peru has significantly contributed to the expansion of knowledge about malaria, particularly in the fields of vivax malaria epidemiology, diagnostics, therapeutics, and vector biology. The contributions include key epidemiological aspects of vivax transmission such as occupation-related travel,^13^ high *P. vivax* parasite genetic diversity,^14^ clustering and microepidemiology of infection,^15^,^16^ the human asymptomatic malaria reservoir,^17^–^19^ some sociodemographic^20^ and microgeographical^21^ characteristics of the sustainability of malaria transmission, and the serious consequences of severe vivax malaria.^22^ In addition, with the discovery of *P. falciparum* strains lacking the histidine-rich protein-2 (PfHRP2)^23^ and the spread of such strains within Peru, researchers were able to assess and identify better alternatives for rapid diagnostic tests (RDTs) specific for the regional strains.^24^ Peru has contributed to clinical research in therapeutics and antimalarial resistance, including a clinical trial of tafenoquine,^25^ and new data on how to improve the utilization of conventional antimalarials within Peru, including mefloquine and artesunate,^26^–^28^ primaquine,^29^,^30^ and dihydroartemisinin–piperaquine.^31^ Furthermore, similar research has contributed to the further understanding of the dynamics of antimalarial resistance, particularly in regards to the resistance to chloroquine,^32^ sulfadoxine–pyrimethamine,^33^–^36^ and mefloquine.^34^ Finally, research from Peru has contributed to the study of vector biology and ecology, particularly on the behavior^37^–^40^ and population structure^41^–^43^ of *Anopheles darlingi* and *Anopheles benarrochi*, the two main malaria vectors in the Amazon basin. We anticipate that as a result of the recently developed model of *An. darlingi* infections,^44^–^46^ the importance of these contributions will only continue to increase. All of these scientific contributions were possible mainly because of a highly productive and internationally collaborative malaria research network that has been established in the Peruvian Amazon and north coast.

## Switching from Control Toward Elimination in Peru: A Work in Progress

**Figure 1.**
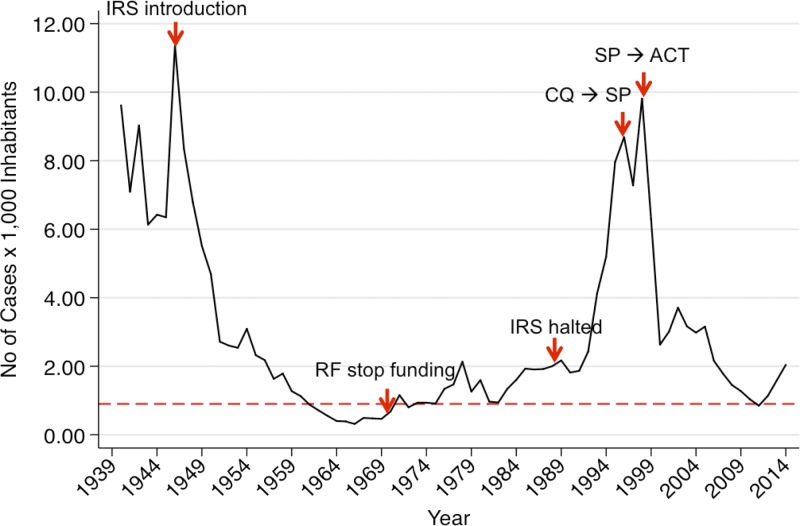
Historical trend of reported malaria incidence in Peru: 1939–2014. Annual Parasite Index (total of subjects that tested smear positive to malaria per 1,000 inhabitants) in Peru since 1939 to date. Here the red dashed line represents the threshold of one case per 1,000 inhabitants, which in the case of Peru has been overcome only twice: in the late 1960s and in the early 2010s. During this whole period, several important interventions were introduced in the country. In 1944, the Rockefeller Foundation sponsored the introduction of dichlorodiphenyltrichloroethane (DDT), but after several years of continuous spraying and malaria-burden decline, such funding ceased in 1970. In 1988, DDT use was halted first in Loreto and later in the rest of the country, mainly because of the emergence of DDT resistance and lack of funding. In 1996, sulphadoxine–pyrimethamine (SP) replaced chloroquine (CQ) as the first-line treatment of uncomplicated falciparum malaria, but after several clinical trials, Peru decided to adopt artemisinin-based combination therapies (ACTs) to replace SP in 2001, remaining as such to date.

In the past, Peru achieved a level of control that twice suggested that malaria elimination might be feasible (Figure 1

). First, after peaking with over 90,000 cases in 1944, the malaria burden fell to below 1,500 cases (98% reduction) in 1965, with ∼1–2 cases per 10,000 inhabitants reported for approximately two decades.^47^ Such success was assumed to be related to the combination of dichlorodiphenyltrichloroethane (DDT) use for vector control, epidemiology and laboratory capacity building, and the shift of focus from case management to transmission control within the National Malaria Service, an effort that was initially sponsored by the Rockefeller Foundation and later by the Inter-American Public Health Service and the United Nations Children's Emergency Fund.^48^ DDT use was halted initially in Loreto in 1988 and then stopped across the country, a policy change that may explain why over the next decade,^49^ malaria increased 4-fold in Peru and 50-fold in Loreto.^47^ After peaking in 1998 with 250,000 cases, the malaria case number dropped to 25,300 cases in 2011, a 90% reduction. The Peruvian authorities quickly responded to this malaria reemergence by updating the national malaria guidelines, introducing mefloquine–artesunate (artemisinin-based combination therapies [ACTs]) as the first line of treatment of *P. falciparum* malaria.^50^,^51^ From 2006 to 2011, Peru implemented the Project of Malaria Control within the Borders of the Andean Region sponsored by the Global Fund. This program implemented a variety of interventions including distributing over 250,000 long-lasting insecticide-impregnated nets (LLIN), using RDTs in remote communities, building capacity for microscopy-based diagnosis, and training community health workers in Loreto only.^52^ We have not considered whether LLINs function effectively in the Amazon, both because of patterns of mosquito feeding and cultural factors. Moreover, previous observations indicate that the use of transparent LLINs was low, mainly because they failed to provide privacy, shelter, and a sense of security for young children as offered by traditional mosquito nets.^53^,^54^ Assessment of peak biting times vis-a-vis sleeping/LLIN usage times remain to be assessed.

**Figure 2.**
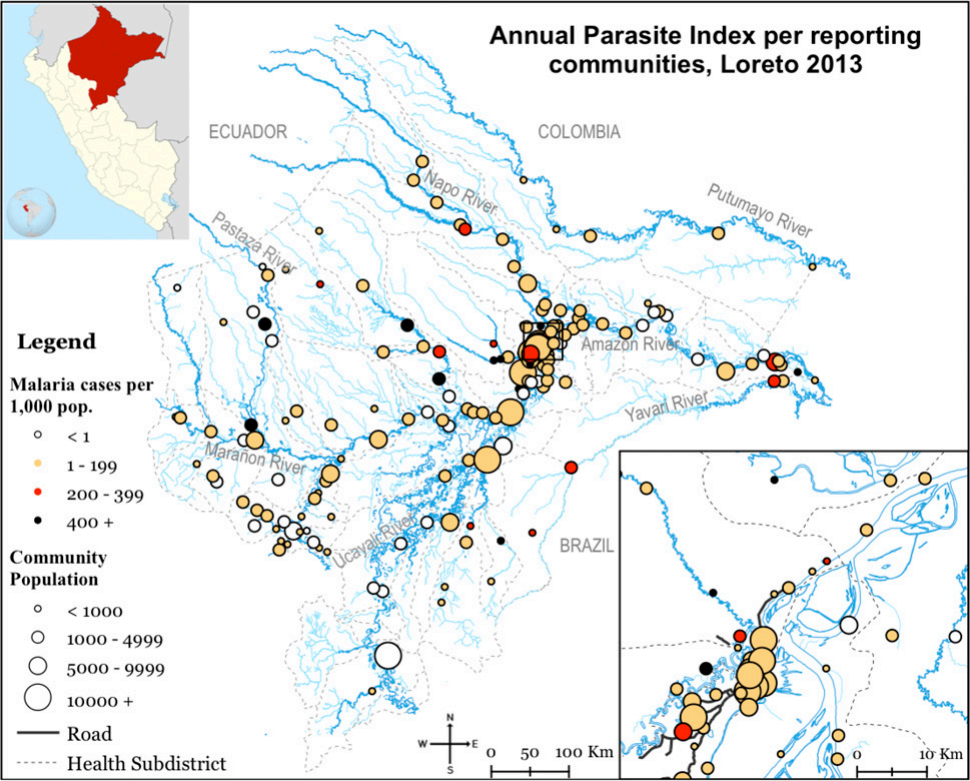
Geographical distribution of reported malaria cases in Loreto, 2013. Distribution of malaria across communities in the Loreto Department. Each bubble represents a reporting community in the national surveillance system. The size of the bubble represents the size of the population of each community, whereas the color represents the Annual Parasite Index (total of subjects that tested smear positive to malaria per 1,000 inhabitants) in the year 2013. The blue lines represent the tributaries of the Amazon river and the dashed line indicates the district boundaries. In 2013, 24% (12/51) of the districts represented over 80% of the total number of malaria cases reported in the Loreto region, whereas 4% of the communities contributed to over 30% of the total number of malaria cases in the same year.

The number of falciparum and vivax malaria cases increased alarmingly from 2,238 to 10,243 and from 9,208 to 52,323 cases, respectively, from 2010 to 2014.^55^ The 2012 flooding and the recent political instability that affected the region are likely contributors to a resurgence of cases. The Amazonian Department of Loreto continues to report the vast majority of malaria cases in Peru at 94%.^55^ In Loreto, malaria is largely seasonal, disproportionally affects adult men (loggers, fishermen, farmers, and other laborers that live in higher transmission regions or travel often^13^,^20^), and is dispersed across riverine communities (Figure 2

).^56^ The epidemiology of malaria in other endemic Amazonian regions such as San Martin and Ucayali vary slightly compared with Loreto. However, in other regions such as on the north coast (Tumbes, Piura, Lambayeque, and La Libertad), the epidemiology is considerably different, occurring with epidemic and sporadic patterns probably related to stochastic factors such as reintroductions.^57^ On the north coast, the predominant malaria vector is *Anopheles albimanus*^58^ and *P. falciparum* malaria has rarely been reported in this region since 2010. Malaria is highly concentrated within periurban communities,^59^ and across the region, the annual number of malaria cases per 1,000 inhabitants has dropped below 0.01 since 2013.^55^ Certainly, the malaria vectors do not stop at any border, neither national nor international, so given that the reintroduction of malaria is likely to occur across the borders, there is a strong need for active program alliances that address both national and transnational issues. Programs such as the Amazon Malaria Initiative and the Peru-Brazil International Centers of Excellence for Malaria Research are contributing to build the necessary knowledge to eliminate malaria in the Peruvian Amazon but more needs to be done.

## Reviewing the Agenda of Malaria Elimination in Peru: Conference Organization and Aims

Early in 2014, a conference was held in the city of Iquitos, the capital of Loreto, on February 16–17 to review the agenda of malaria elimination in Peru. The main objective was to engage the research community and decision makers in a collaborative effort to define the critical knowledge gaps and policy requirements to achieve and sustain malaria elimination in Peru. In attendance were representatives from regional organizations, national organizations, the Ministry of Health, and international participants. In this conference, 25 different organizations were represented (Table 1). Local experts and stakeholders were well represented in the conference. Of the 85 conference participants, 53 were Peruvian, including 34 researchers and authorities from the Loreto region, and 32 were international, most with long track records working on national public health priorities in Peru. Four of the six board members were Peruvian and 19 of the 30 consulting committee members were Peruvian.

To meet this objective, the conference integrated two formats for soliciting expert input and achieving consensus. On the first day, individual experts gave presentations that reviewed: past malaria control and elimination efforts; current opportunities and challenges for malaria elimination in the Americas; the potential role of transmission-blocking vaccines, seroepidemiology, and diagnostic technologies in malaria elimination; and the role of social and behavioral considerations in malaria diagnostics and interventions. The discussions included the microepidemiology of malaria, various strategies and challenges of malaria elimination in this region, and the benefits and practicalities of a demonstrative elimination project. On the second day, we convened the first meeting of the newly formed Malaria Elimination Working Group, a multiorganizational, multidisciplinary group of malaria researchers convened to support the agenda of malaria elimination in the Amazon region, with a particular focus in the Peruvian Amazon. The group's goal is to promote innovative solutions to the scientific, technical, operational, financial, and programmatic challenges that decision makers will need to address when pursuing a malaria elimination initiative. The second day's agenda comprised four different round table discussions led by experts to achieve consensus on several different aspects that were considered highly relevant to malaria elimination. These included: 1) feasibility and project sites, 2) project aims and outcomes, 3) diagnostics, interventions, and methods, and 4) the gaps and priorities in the future research agenda. During the closing discussion of the conference, the moderators of each group presented the outcomes of their discussions.

## Feasibility of a Malaria Elimination Project in the Peruvian Amazon

The group concluded that implementing a malaria elimination project in the Peruvian Amazon is feasible, although its success will require a clear financial and political commitment from the Peruvian government. For this purpose, it will be crucial to establish a comprehensive regional strategic plan to guide this project. Peruvian public health activities should continue to focus on basic prevention and control and to introduce new tools and new strategies as they are developed. The implemented measures must be integrated, culturally appropriate, and sustainable in terms of both financing and political support. The incorporation of other regional and local authorities (such as provincial governors and local leaders at the city and village levels) will also be important. A critical aspect of the strategy will be the identification of asymptomatic parasitemic individuals, who are important reservoirs for continuing transmission, and monitoring how subsequent interventions affect this population's potential for maintaining local malaria transmission. Targeted parasite elimination strategies that are appropriate to the region must be used to overcome the limitations of passive case detection and the difficulties in providing proper coverage to the isolated hard-to-reach populations (either remote communities or migratory subpopulations such as loggers and gold miners). Several criteria should be considered in the selection of study sites: they should have a large population with low annual parasite index (API < 1) and cultural and political accessibility. The setting must allow for the incorporation of new validated tools to augment standard control measures. In addition, the study sites must be close to each other, potentially within the same river basin, and the regions surrounding the study area must have control measures in place to prevent reintroduction. Finally, considering the ethical implications, it must be determined whether these interventions are in the public health interest of the study sites, and if successful, these interventions should be scaled up to include neighboring communities.

## Aims and Outcomes of a Malaria Elimination Project

The group discussed various outcomes that might be useful in assessing the impact of a malaria elimination strategy in the Peruvian Amazon. Many indicators were evaluated but only a few were recommended by the group to be considered relevant to measure the impact of a malaria elimination project. These included the following: burden of disease, API, positivity index, incidence rate, seroprevalence, population structure of the vector species, human biting rate, sporozoite index, and vector density. Furthermore, the group stressed the relevance of adapting or developing some process indicators. These indicators should at least allow a proper measurement of the project coverage, community responsiveness, capacity building, as well as the actions to convert the project inputs into specific human behavior changes. Consensus could not be reached regarding a reasonable time frame within which malaria elimination could be achieved in the study sites, primarily because of uncertainties of funding and variations on approaches. However, the group agreed on several themes that will be essential when considering malaria elimination as the ultimate goal. These include an integrated strategy with properly financed, independent, and dedicated resources and the subsequent integration of the proposed diagnostic and intervention measures into the health system. Also, it will be essential to improve logistic communication and programmatic monitoring activities among involved interdisciplinary and intersectoral groups. In the scope of a malaria elimination project, there was a strong agreement that it would be critical to involve public health and political authorities at the highest political level to achieve programmatic stability.

## Key Aspects of Diagnostics, Interventions, and their Implementation in the Peruvian Amazon

Participants in this roundtable carried out a lively discussion on the need for improving current regional diagnostic platforms with more sensitive but portable diagnostics, particularly for targeting rural communities and hard-to-reach high-risk populations such as loggers, farmers, and fishermen. In this discussion, the group emphasized that it will be critical to introduce sufficiently sensitive RDTs that are appropriate for the Peruvian context as well as to address the administrative and logistical barriers that might prevent a regular and continuous supply. This is particularly true for both *P. falciparum* and *P. vivax*, given the high prevalence of PfHRP2,^23^ its multiple genetic origins,^24^ and the considerable variability in the diagnostic accuracy of some of the pan-lactate dehydrogenase–detecting RDTs in the Peruvian Amazon.^60^ In addition, its implementation should follow locally adapted guidelines. The group recommended that interventions should be implemented as an integrated package, accounting for the local sociocultural barriers to classical interventions such as bed nets and indoor residual spraying and placing interventions into a local context to maximize impact. It will be essential to monitor and evaluate programs and interventions from a research perspective (e.g., surveillance for emerging drug resistance). Consensus was reached on the need to have a mechanism to maintain updated Peruvian national malaria clinical guidelines; for example, taking into account new data indicating the need for ACT plus primaquine to effectively treat and block transmission of *P. falciparum* malaria. Moreover, to shift the goal from control toward elimination, there is a need to target the malaria parasite reservoir (treating both asymptomatic and symptomatic infections) instead of just the malaria disease burden (malaria symptomatic infections only). Finally, for the initiative to be successful, the group agreed that steps must be taken to assure a continual supply of the RDTs and antimalarial drugs proposed for use in the region.

## Gaps and Priorities in the Future Research Agenda

Several recommendations were made regarding key elements of the future research agenda (Table 2). These include that research and evaluation of a malaria elimination project should have clear and achievable milestones, progress from simple to more complex interventions, and involve all the different stakeholders (national and local government, academia, and nongovernmental organizations). Its implementation should be carefully planned to overcome operational challenges by identifying and integrating key providers within the project. Mobile and internet coverage is currently restricted mainly to periurban Iquitos, which represents a major barrier for the development of public health interventions. The implementation and operational research should incorporate social science and account for the social determinants of malaria to find culturally acceptable ways to intervene. Integrated approaches should be considered as means to strengthen the impact of any malaria elimination project, particularly in the most remote communities, as well as to sustain the political will and support. Importantly, the focus of elimination efforts must be on the most important reservoirs of maintaining malaria endemicity and transmission rather than solely on symptomatic individuals. Such an approach will require better tools to efficiently identify carriers (serology and molecular assays complementary to RDTs and microscopy). Research on vector behavior, including monitoring and surveillance for insecticide resistance, as well as research on the local customs and preferences for bed net usage are crucial for understanding and deploying effective and widely used vector control interventions. With regards to therapeutics, there is a need for improved policies to establish a streamlined process to incorporate new drugs into the Peruvian national formulary.

## Conclusions and Future Directions

Several key conclusions and points of consensus arose from this meeting. The most important one is that malaria elimination in the Peruvian Amazon can be achievable and should be a national and international priority. A comprehensive regional strategic plan needs to be developed that addresses key technical, operational, and economical considerations such as the ones recommended by the World Health Organization. It was strongly recommended to first pilot such a strategy in suitable sites in the region to establish efficacy and acceptability. When such a strategy is implemented, it will be important to monitor and evaluate progress through a variety of metrics and to set intermediate goals on the path to regional elimination. Targeted parasite elimination strategies that are appropriate to the region must be used, stressing active case detection using sufficiently sensitive and effective RDTs and species-specific treatment of the asymptomatic reservoir. This is particularly important in the case of *P. falciparum* malaria, which must be treated with ACT and primaquine to interrupt transmission. The strategy must facilitate the communication between key stakeholders from the region and political support at all levels of government, and the program should be incorporated into established health-care systems to improve acceptability and sustainability. The progression of such a strategy should be flexible to allow new knowledge of the social determinants of malaria, the cultural acceptability of key interventions, and novel tests and treatments to be incorporated throughout the effort. An agreement on the relevance of pursuing malaria elimination as a goal was reached during the conference, and the necessary components and characteristics of this effort were described. Moving forward, further details should be elaborated as commitments from a critical mass of stakeholders are obtained.

## Figures and Tables

**Table 1:** Partners and collaborating organizations

Acronym	Organization name
BMGF	Bill & Melinda Gates Foundation
CGHD-CWRU	Case Western Reserve University
CSRC	Caucaseco Scientific Research Center
DGE	Dirección General de Epidemiología (General Epidemiology Directorate)
DIRESA Junín	Dirección Regional de Salud de Junín (Junin Regional Health Directorate)
DIRESA Loreto	Dirección Regional de Salud de Loreto (Loreto Regional Health Directorate)
DIRESA Madre de Dios	Dirección Regional de Salud de Madre de Dios (Madre de Dios Regional Health Directorate)
DIRESA Piura	Dirección Regional de Salud de Piura (Piura Regional Health Directorate)
DIRESA San Martín	Dirección Regional de Salud de San Martín (San Martin's Regional Health Directorate)
DIRESA Tumbes	Dirección Regional de Salud de Tumbes (Tumbes' Regional Health Directorate)
ESNEM	Estrategia Sanitaria Nacional de Prevención y Control de Enfermedades Metaxenicas (National Sanitary Strategy for the Prevention and Control of Vector Borne Diseases)
GRL	Gobierno Regional de Loreto (Loreto Regional Government)
ICEMR	Peru–Brazil International Center of Excellence in Malaria Research
INS	Instituto Nacional de Salud (National Health Institute)
JHBSPH	Johns Hopkins Bloomberg School of Public Health
MINSA	Ministerio de Salud (Ministry of Health)
NAMRU-6	U.S. Naval Medical Research Unit No. 6
PAHO	Pan American Health Organization
PATH	Program for Appropriate Technology in Health
PRISMA	Proyectos de Informática, Salud, Medicina, y Agricultura
UCSD	University of California, San Diego
UNAP	Universidad Nacional de la Amazonía Peruana, Loreto (National University of the Peruvian Amazon)
UPCH	Universidad Peruana Cayetano Heredia (Cayetano Heredia Peruvian University)

**Table 2:** Summary points of consensus

Malaria elimination in the Peruvian Amazon is a feasible and important goal, but will require:
1. A comprehensive regional strategic plan and integrative control measures that are culturally and contextually appropriate as well as politically and financially sustainable.
2. Study sites for new control measures should have large populations, low transmission, and be accessible but resistant to contamination of effect.
3. Evaluation of four types of indicators: morbidity/mortality, surveillance/monitoring, entomological, and process indicators.
4. That rapid diagnostic tests (RDTs) be introduced for active case detection, ideally with local production at some point, and that microscopy should be maintained while its quality is improved.
5. Interventions that prioritize active detection with RDTs and treatment with artemisinin-based combination therapy with primaquine over bed nets and spraying, which have limited effectiveness in this setting, while establishing a continual supply chain for tests and antimalarial drugs.
6. Research to address knowledge gaps in vector behavior, insecticide resistance, the social determinants of malaria, and cultural perspectives toward particular interventions, while working to develop and implement novel assays for detection and therapeutics.
7. Integration of the program into the existing health system, a communication platform between stakeholder groups, and intersectoral collaboration between stakeholders and officials at all levels of government.
